# Human Monocyte-Derived Osteoclasts Are Targeted by Staphylococcal Pore-Forming Toxins and Superantigens

**DOI:** 10.1371/journal.pone.0150693

**Published:** 2016-03-02

**Authors:** Sacha Flammier, Jean-Philippe Rasigade, Cédric Badiou, Thomas Henry, François Vandenesch, Frédéric Laurent, Sophie Trouillet-Assant

**Affiliations:** 1 Centre International de Recherche en Infectiologie, INSERM U1111, Pathogenesis of staphylococcal infections, University of Lyon 1, Department of Clinical Microbiology, Northern Hospital Group, Hospices Civils de Lyon, 103 grande rue de la Croix Rousse, 69004 Lyon, France; 2 Centre International de Recherche en Infectiologie, INSERM U1111, Inflammasome, bacterial infections and autoinflammatory diseases, University of Lyon 1, 21 avenue Tony Garnier, 69365 Lyon cedex 07, France; Faculté de médecine de Nantes, FRANCE

## Abstract

*Staphylococcus aureus* is the leading cause of bone and joint infections (BJIs). Staphylococcal pathogenesis involves numerous virulence factors including secreted toxins such as pore-forming toxins (PFTs) and superantigens. The role of these toxins on BJI outcome is largely unknown. In particular, few studies have examined how osteoclasts, the bone-resorbing cells, respond to exposure to staphylococcal PFTs and superantigens. We investigated the direct impact of recombinant staphylococcal toxins on human primary mature monocyte-derived osteoclasts, in terms of cytotoxicity and cell activation with cell death and bone resorption assays, using macrophages of the corresponding donors as a reference. Monocyte-derived osteoclasts displayed similar toxin susceptibility profiles compared to macrophages. Specifically, we demonstrated that the Panton-Valentine leukocidin, known as one of the most powerful PFT which lyses myeloid cells after binding to the C5a receptor, was able to induce the death of osteoclasts. The archetypal superantigen TSST-1 was not cytotoxic but enhanced the bone resorption activity of osteoclasts, suggesting a novel mechanism by which superantigen-producing *S*. *aureus* can accelerate the destruction of bone tissue during BJI. Altogether, our data indicate that the diverse clinical presentations of BJIs could be related, at least partly, to the toxin profiles of *S*. *aureus* isolates involved in these severe infections.

## Introduction

Bone is a mineralized tissue in a constant renewal process called bone remodelling, provided by the coordinated action of two main cell types, osteoblasts, the bone-forming cells, and osteoclasts, the bone-resorbing cells [1]. « Osteoblasts derive from mesenchymal stems, while osteoclasts have a myeloid origin and share common features with macrophages including a phagocytic activity [2]. Osteoclast maturation involves the fusion of several mononucleated osteoclast precursors into giant multinucleated-cells endowed with the bone-matrix resorption ability. In a physiological context, bone integrity is maintained by a balance between osteoblastic and osteoclastic activities throughout life. During bone and joint infections (BJIs), including osteomyelitis and orthopaedic device infections, this process is impaired by the interaction of bacteria with bone tissue, leading to bone destruction [3]. Indeed, *Staphylococcus aureus*, the leading cause of BJIs, is responsible for bone infections marked by progressive bone loss [4].

Numerous studies have investigated the direct impact of *S*. *aureus* on osteoblasts [5],[6]. It is well-known that this pathogen is able to adhere to osteoblasts, become internalized and survive intracellularly and/or induce cell death [7]. Moreover, several studies have demonstrated the ability of live *S*. *aureus* to inhibit osteoclastogenesis and to increase bone resorption mediated by osteoclasts [2]. These observations suggest that *S*. *aureus* directly interacts with bone cells, modifying their functions of bone mineralization or bone resorption. Nevertheless, the pathophysiologic mechanisms, responsible for the bone destruction observed in BJI caused by *S*. *aureus*, remain incompletely understood.

*S*. *aureus* is able to act on remote target cells through secreted virulence factors, including toxins. *S*. *aureus* expresses a large panel of pore-forming toxins (PFTs) that target the host cell membrane including α- (Hla), β- (Hlb), γ- (HlgAB and HlgBC) haemolysins, and leukocidins (LukED, LukGH and the Panton Valentine Leukocidin [PVL]). PFT-induced permeabilization of the cytoplasmic membrane results in the efflux of intracellular metabolites and ultimately cell death [8]. *S*. *aureus* also expresses superantigenic toxins such as the toxic shock syndrome toxin (TSST-1) or staphylococcal enterotoxins (SEA, SEB, etc.) responsible for a polyclonal activation and a massive proliferation of T cells independent of antigen specificity. Most of these staphylococcal toxins target immune cells derived from the myeloid lineage (monocytes, macrophages and dendritic cells), a characteristic which is thought to help *S*. *aureus* escape the immune system [9].

Noteworthy, several of the aforementioned toxins exhibit some degree of specificity with respect to immune cells through the specific binding of cell surface receptors [10]. Because osteoclasts derive from progenitors of the myeloid lineage, we hypothesized that their susceptibility to staphylococcal toxins share some similarities with the susceptibility of other myeloid cells such as macrophages, which could be of interest for our understanding of the pathophysiology of *S*. *aureus* BJIs. We thus tested the direct effect of a panel of recombinant staphylococcal toxins on primary human mature osteoclasts by assessing cell cytotoxicity. We also investigated the impact of the TSST-1 superantigen on the bone resorption activity of osteoclasts.

## Materials and Methods

### Preparation of osteoclasts

Monocytes were purified from the blood of healthy donors (n = 3) purchased from Etablissement Français du Sang (Lyon Gerland, France), as previously described [11]. Donors gave written consent to EFS for the use of blood sample for research purposes at the time of sampling (number of the agreement linking EFS and the research laboratory: 14-1820-69). Briefly, after collection, blood was loaded on a Lymphocyte Separation Medium density gradient (Eurobio, Courtaboeuf, France) to purify mononuclear cells. Cells were then centrifuged through a 50% Percoll density gradient to concentrate monocytes and purified from the light-density fraction by immunomagnetic depletion using magnetic beads (Dynabeads goat anti-mouse IgG, Invitrogen, Carlsbad, CA) and a cocktail of monoclonal antibodies (mAbs): anti-CD19, anti-CD3, anti-CD56 and anti-glycophorin A (Beckman-Coulter, Miami, FL), ensuring purification rates ≥ 95%. Monocytes were then plated in 96-well plates at a density of 10^5^cells/well and cultured in α-MEM medium (Gibco Life Technologies, Inc., Grand Island, NY) supplemented with 2 mM L-glutamine (Gibco Life Technologies), 1% penicillin/streptomycin (Gibco Life Technologies), and 10% foetal bovine serum (PAN Biotech, Aldenbach, Germany).

Monocytes were differentiated into mature osteoclasts or macrophages as described elsewhere [11]. Briefly, osteoclasts were obtained by incubating monocytes with 50 ng/mL M-CSF (Monocyte Colony-stimulating Factor) (PeproTech, Rocky Hill, NJ) and 30 ng/mL of RANKL (Receptor Activator of Nuclear factor Kappa-B Ligand) (PeproTech, Rocky Hill, NJ) from day 1 to day 3, and in presence of 25 ng/mL of M-CSF and 100 ng/mL of RANKL from day 3 to day 6. Macrophages were obtained by incubation of monocytes with 50 ng/mL of M-CSF only from day 0 to day 6.

### Toxins production

Recombinant staphylococcal toxins (Hla, Hlb, HlgAB, HlgBC, LukED, LukGH, PVL, TSST-1 and SEA) were purified as described elsewhere [12]. The endotoxin content of the recombinant protein solutions was controlled and confirmed to be less than 0.004 endotoxin units per μg of protein.

### Cytotoxicity assay

Toxin cytotoxicity to osteoclasts and macrophages was quantified by monitoring propidium iodide (PI) incorporation as previously described [13]. Cells were incubated with PI (5 μg/ml) and variable concentrations of recombinant staphylococcal toxins. Propidium iodide fluorescence was measured over a 3-hour period on a microplate fluorimeter (Tecan, Lyon, France), using untreated cells as control.

### Bone resorption assay

Bone resorption was quantified as a means to assess the impact of the TSST-1 superantigen on osteoclast activity. On day 6, mature osteoclasts were detached from plastic wells by flushing after incubation with Accutase (Invitrogen Life Technologies, Gaithersburg, MD) (37°C-30 min), and then seeded at 2.10^4^ cells/well on mineralized matrix Osteo Assay Surface 96-well plates (OsteoCorning®, Corning, MA, USA). TSST-1 was added to wells at 0.1, 1, 10, 100 and 1000 ng/ml. Twenty four hours later, osteoclasts were lysed by osmotic shock, and OsteoCorning® Assay plates were stained with PBS/5% silver nitrate (Sigma-Aldrich) to quantify resorption using a Leica 22 DMI6000 microscope (Nanterre, France) and Fiji software (US National Institutes of Health, Bethesda, Maryland, USA) as described elsewhere [14]. Results were expressed as the proportion of resorbed area in each condition relative to the resorbed area in untreated cells.

### Statistical analyses

Differences in means were analysed using Student’s test (t-test) with a threshold of 0.05. Analyses were performed using R software, version 2.14.2 (The R foundation for statistical computing, Vienna, Austria). Results were expressed as means and 95% confidence intervals derived from three independent experiments realized in triplicate. Each experiment was realized with a different blood donor.

## Results

**Fig 1:**
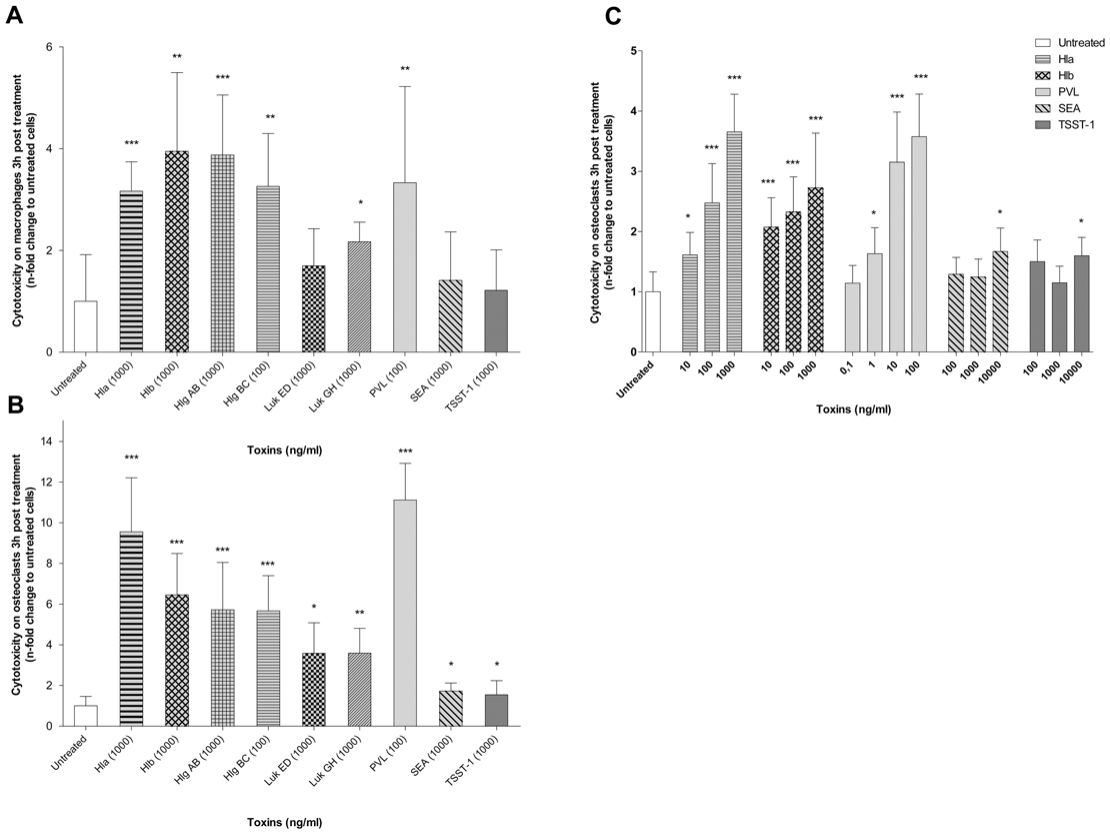
Cytotoxicity of staphylococcal toxins on human macrophages and mature osteoclasts. Human monocytes were differentiated for 6 days into macrophages with macrophage colony-stimulating factor (M-CSF) (A) or into mature osteoclasts in the presence of M-CSF and receptor activator of NFκ-B ligand (RANK-L) (B and C). Staphylococcal toxins were then added to the cell culture medium containing propidium iodide (PI) and cell death was quantified by monitoring PI incorporation over a 3 hours period (A, B and C). Fluorescence in each well was normalised to the fluorescence obtained with untreated cells. Results represent the mean cytotoxicity with 95% confidence interval, of 3 independent experiments performed in triplicate on 3 different donors (*p<0.05, ** p<0.01, ***p<0.001). Hla: α haemolysin, Hlb: β haemolysin, Hlg AB and Hlg BC: γ haemolysins AB and BC, Luk ED and Luk GH: leukocidins ED and GH, PVL: Panton Valentine Leukocidin, TSST-1: toxic shock syndrome toxin, SEA: Staphylococcal enterotoxin A.

We first evaluated the cytotoxic effect of a wide range of staphylococcal toxins on human macrophages by measuring incorporation of PI 3 hours post treatment. Macrophages served as a reference profile of the different toxins’ activities (Fig 1). As expected, we observed a high cytotoxicity of the membrane-damaging toxins (Hla, Hlb, HlgAB, HlgBC, LukGH and PVL) to human macrophages while superantigenic toxins did not cause significant cytotoxicity compared to untreated cells. These results, consistent with those obtained in literature [12], were used to validate our experimental protocol.

Because osteoclasts share a common myeloid origin with macrophages, we hypothesized that staphylococcal toxin cytotoxic activity profiles on osteoclasts could be similar with those observed with macrophages. To confirm this hypothesis, the experimental protocol used with macrophages was tested on mature human osteoclasts (Fig 1). Results indicated that pore-forming toxins induced a significant cytotoxicity on mature human osteoclasts unlike superantigens. Moreover, cytotoxicity profiles appeared to be superimposable between macrophages and osteoclasts. Toxin-induced cytotoxicity to macrophages and osteoclasts could only be compared qualitatively (cytotoxicity profiles caused by the various toxins), and not quantitatively (Fig 1). Indeed, mature osteoclasts have dozens of nuclei so this increased DNA content per cell leads to higher values of PI incorporation-induced fluorescence as compared to macrophages, which prevented us to compare fluorescence values of macrophages and osteoclasts.

Using increasing concentrations of toxins, we showed that membrane-damaging toxins had a dose-dependent cytotoxic effect on mature human osteoclasts. For example PI incorporation in cells treated with PVL at 0.1, 1, 10 and 100 ng/ml were respectively 14%, 63%, 315% and 358% higher, compared to untreated (Fig 1). In contrast, superantigenic toxins caused significant cytotoxicity on osteoclasts only above 10 000 ng/mL.

**Fig 2:**
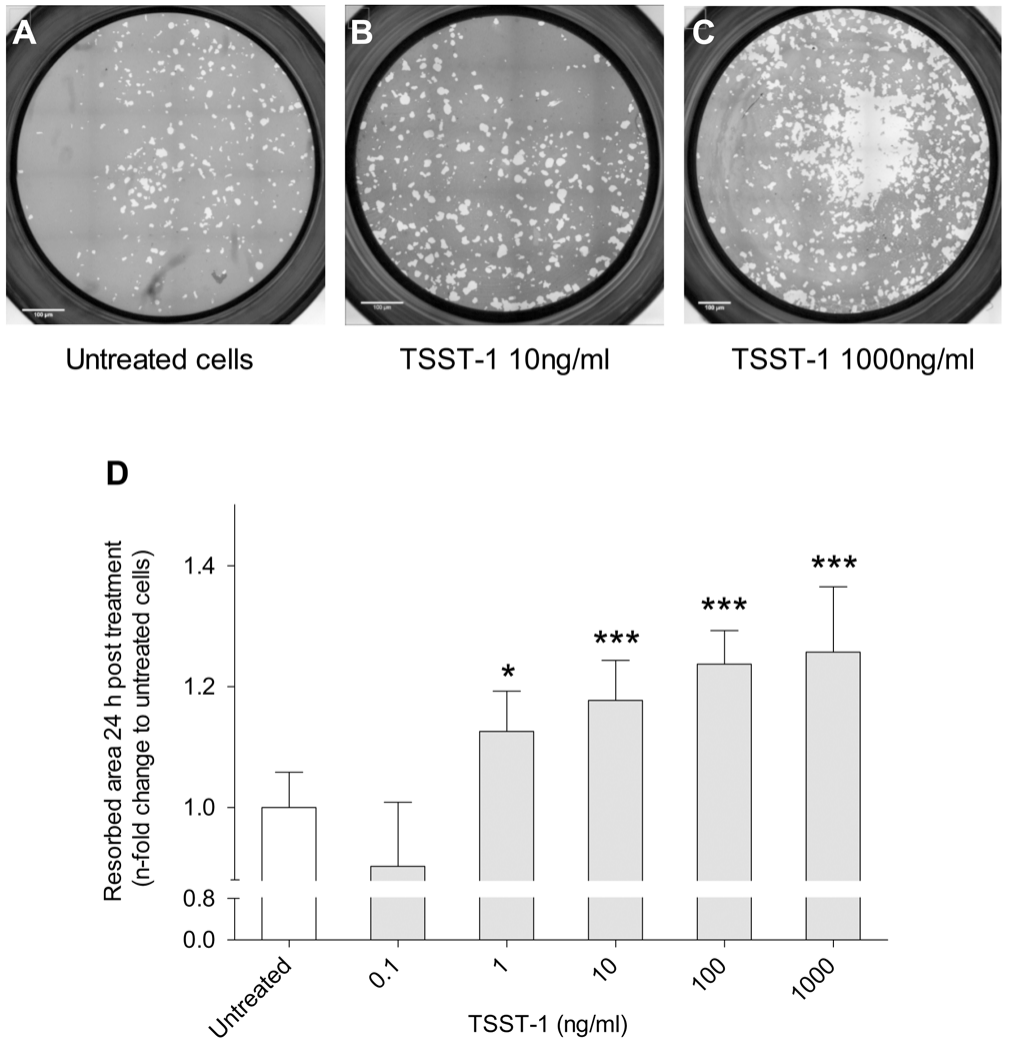
TSST-1 stimulates bone resorption capacity of mature human osteoclasts. For bone resorption assay, mature osteoclasts were detached from plastic on day 6 and seeded at 2.10^4^ cells/well on mineralized matrix Osteo Assay Surface 96-well plates. TSST-1 was added to the cell culture medium. After 24 hours of culture, the osteocorning matrices were stained with 5% silver nitrate to measure the resorbed area (white area). The percentages of matrix that were resorbed by untreated (A) or TSST-1-treated osteoclasts (B, C) were measured using the Fiji software. Bars represent 100 μm. Results of resorption area quantification (D) represent the mean of resorbed area with 95% confidence interval, of 3 independent experiments realised in triplicate on 3 different donors (*p<0.05, ** p<0.01, ***p<0.001). TSST-1: Toxic shock syndrome toxin.

Because it has been established that some staphylococcal toxins also have cellular activation effect [15], we tested the impact of the non-cytotoxic toxins on osteoclast activation. We investigated the capacity of TSST-1 to activate mature human osteoclasts by assessing bone resorption assays (Fig 2). Results demonstrated that TSST-1 significantly enhanced osteolytic activity in a dose-dependent manner, resorbed area by cells treated with TSST-1 at 1, 10, 100, 1000 ng/ml were respectively 13%, 17%, 24% and 26% higher, compared to untreated cells (p<0.001 for all). TSST-1 concentrations below 1 ng/mL induced no measurable effect.

## Discussion

Using an *in vitro* model, we evaluated the direct, specific, and independent effect of recombinant *S*. *aureus* toxins on mature human monocyte-derived osteoclasts. Using osteoclasts and macrophages differentiated from the same blood donors, we have shown that human macrophages and mature monocyte-derived osteoclasts exhibit similar susceptibility profiles with respect to staphylococcal toxins. PFTs caused significant dose-dependent cellular cytotoxicity on these two cell types whereas superantigenic toxins were not or poorly cytotoxic. Noteworthy, these results are in agreement with the fact that mature osteoclasts express the complement receptor C5a [16], which has recently been identified as the PVL and HlgBC receptor [17]. Moreover, several studies have demonstrated that human osteoclasts express CCR2, CCR5 and CXCR1 and 2 [18], [19], [20] which have recently been identified as receptors respectively of LukED, HlgBC and both of these PFTs [10]. This suggests that osteoclasts are targeted by PVL, HlgBC and LukED during BJIs. The direct cytotoxic activity of PVL highlighted in this study could play a role, which level remains to be determined, in the severity and outcome of acute BJIs due to PVL-producing *S*. *aureus*. Indeed, it is known that BJIs caused by *S*. *aureus* PVL positive strains are more severe and extensively destructive than those caused by *S*. *aureus* PVL negative ones [21]. The direct effect on osteoclasts could be added to the cytolytic indirect effect related to the release, at the infection site, of the cytoplasmic content of macrophages and neutrophils, which were the only target cells previously identified for PVL. Previous studies have demonstrated that PVL, in addition to targeting neutrophils can target macrophages and to trigger IL-1β secretion [15]and amplify inflammation. This content has likely a direct cytotoxic effect on osteoblasts, osteoclasts and the bone tissue itself, leading to a local inflammation, tissue necrosis and thus to bone destruction.

As superantigens were not cytotoxic to osteoclasts, the second part of this study aimed to assess the ability of TSST-1, to stimulate mature monocyte-derived osteoclasts. It has been shown that staphylococcal superantigens ensure, in absence of antigen presentation, bridging between the TCR Vβ chain of T cells and MHC class II of antigen presenting cells and in particular by osteoclasts [22]. Although the presence of these two cell types appears to be required for the synergistic action of superantigens, several studies have reported the ability of superantigens to stimulate macrophage pro-inflammatory cytokine secretion in the absence of T cells [23]. Our data show that TSST-1 promotes bone resorption by human mature monocyte-derived osteoclasts at concentrations above 1 ng/mL, which are probably relevant in the context of bone infection *in vivo*. Indeed, plasmatic TSST-1 concentrations of more than 5 ng/mL have been observed in infected patients [24], and it is likely that toxin concentrations at the site of infection are greater than those in circulating blood. Importantly, *S*. *aureus* strains can harbor other superantigens than TSST-1. Whether these superantigens could trigger osteoclastogenesis similar to TSST-1 remains an open question. Indeed, the osteoclastic stimulation observed in our model might be specific to TSST-1, because this toxin has been shown to interact with several cell surface targets including ADAM17 and EGFR [25] or CD40 [26], which are expressed by osteoclasts [27][28][29]. Further studies are warranted to determine which osteoclastic receptors are involved in TSST-1-induced stimulation, and to determine whether this stimulation involves a canonical superantigen-MCH class II interaction which might be triggered by other superantigens.

Collectively, our results suggest that bone loss during staphylococcal BJIs might not only be driven by non-specific inflammation and local acidity, created by dead cells, but also by the specific targeting and activation of bone resorbing cells by bacterial toxins. The balance between osteoclasts killing by PFTs and the superantigen-mediated increase in osteoclasts’ bone resorption activity may control the different clinical expression of BJIs associated with the toxinic profile of the different *S*. *aureus* strains.
